# Crystallographic Study of Peptidoglycan Biosynthesis Enzyme MurD: Domain Movement Revisited

**DOI:** 10.1371/journal.pone.0152075

**Published:** 2016-03-31

**Authors:** Roman Šink, Miha Kotnik, Anamarija Zega, Hélène Barreteau, Stanislav Gobec, Didier Blanot, Andréa Dessen, Carlos Contreras-Martel

**Affiliations:** 1 University of Ljubljana, Faculty of Pharmacy, Aškerčeva 7, Ljubljana, Slovenia; 2 Lek Pharmaceuticals d. d., Verovškova 57, Ljubljana, Slovenia; 3 Laboratoire des Enveloppes Bactériennes et Antibiotiques, Institut de Biologie Intégrative de la Cellule (I2BC), CEA, CNRS, Université Paris-Sud, Gif-sur-Yvette, France; 4 Univ. Grenoble Alpes, Institut de Biologie Structurale, Grenoble, France; 5 CNRS, IBS, Grenoble, France; 6 CEA, IBS, Grenoble, France; 7 Brazilian National Laboratory for Biosciences (LNBio), CNPEM, Campinas, São Paulo, Brazil; Institut Pasteur Paris, FRANCE

## Abstract

The biosynthetic pathway of peptidoglycan, an essential component of bacterial cell wall, is a well-recognized target for antibiotic development. Peptidoglycan precursors are synthesized in the bacterial cytosol by various enzymes including the ATP-hydrolyzing Mur ligases, which catalyze the stepwise addition of amino acids to a UDP-Mur*N*Ac precursor to yield UDP-Mur*N*Ac-pentapeptide. MurD catalyzes the addition of D-glutamic acid to UDP-Mur*N*Ac-L-Ala in the presence of ATP; structural and biochemical studies have suggested the binding of the substrates with an ordered kinetic mechanism in which ligand binding inevitably closes the active site. In this work, we challenge this assumption by reporting the crystal structures of intermediate forms of MurD either in the absence of ligands or in the presence of small molecules. A detailed analysis provides insight into the events that lead to the closure of MurD and reveals that minor structural modifications contribute to major overall conformation alterations. These novel insights will be instrumental in the development of new potential antibiotics designed to target the peptidoglycan biosynthetic pathway.

## Introduction

The development of bacterial resistance to antibiotics worldwide is a phenomenon that urges the need to discover new antibacterial drugs. The biosynthetic pathway of peptidoglycan, a key component of the bacterial cell wall, is a tractable target since proteins that catalyze key reactions in the pathway can be targeted both by natural and synthetic antibiotics. [1–4] Peptidoglycan is a complex heteropolymer that consists of glycan chains cross-linked by short peptides. [5] The glycan chains are made up of alternating *N*-acetylglucosamine (Glc*N*Ac) and *N*-acetylmuramic acid (Mur*N*Ac) residues linked by β1→4 bonds. The lactoyl group of the Mur*N*Ac residues is substituted by a peptide stem most commonly composed of L-Ala-γ-D-Glu-*meso*-A_2_pm (or L-Lys)-D-Ala-D-Ala (A_2_pm, 2,6-diaminopimelic acid). Cross-linking of the stem peptides generally occurs between the carboxyl group of D-Ala at position 4 and the amino group of the diaminoacid at position 3, either directly or through a short peptide bridge. [5]

**Fig 1:**
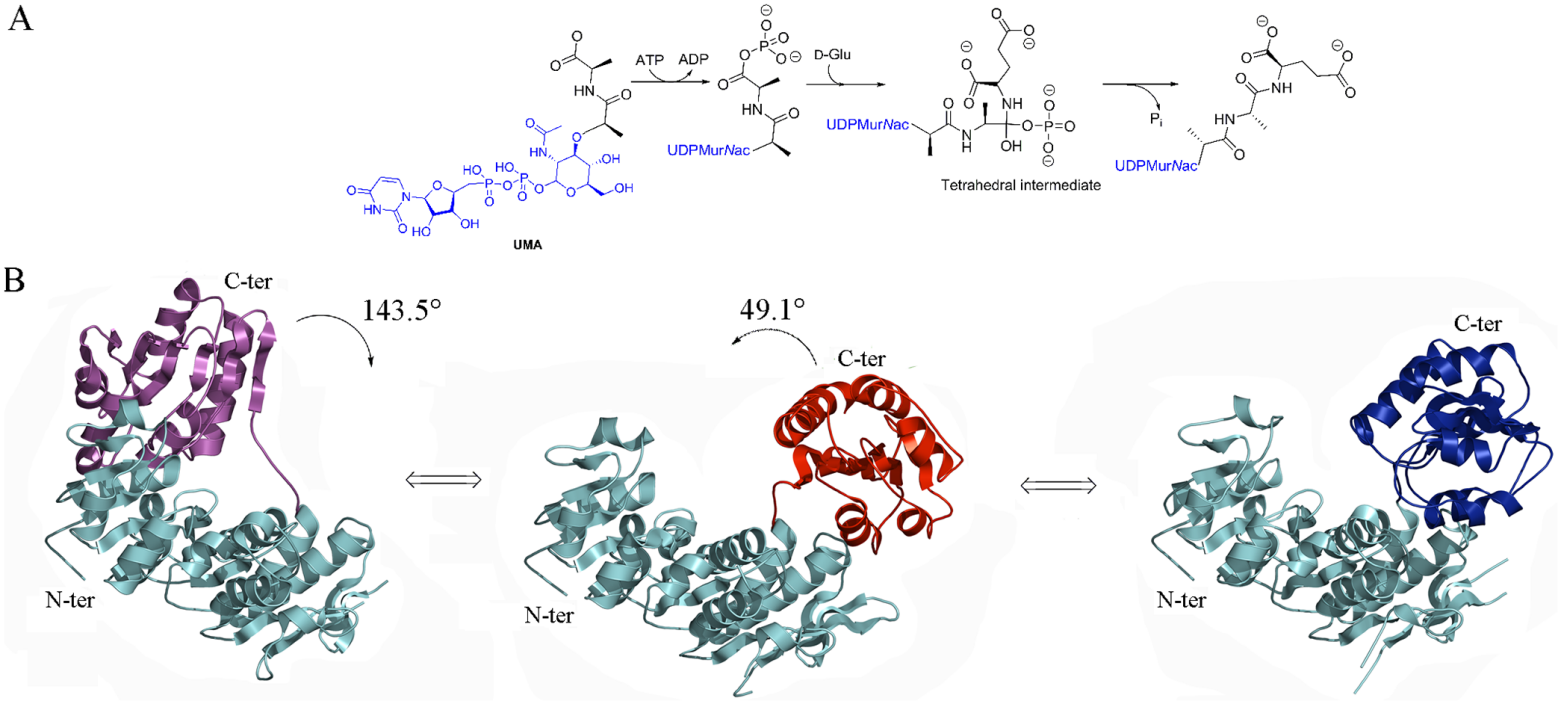
Reaction mechanism and known conformations of MurD. (A) Mechanism of reaction catalyzed by MurD. (B) Rotation of the C-terminal domain from Open UMA-bound (Magenta) (PDB entry: 1eeh [7]) to Open (Red) (PDB entry: 1e0d [7]) to Closed (Blue) (PDB entry: 3uag [6]) with identified angles of rotation. For the sake of clarity N-terminal and central domains are colored in cyan in all conformations.

The biosynthesis of peptidoglycan can be roughly divided into three stages: cytoplasmic, membrane-associated, and periplasmic. Within the bacterial cytoplasm, Mur ligases perform the stepwise addition of amino acids to UDP-*N*-acetylmuramic acid (UDP-Mur*N*Ac), thereby building a stem peptide. Mur ligases require ATP for activity, and are absolutely essential for bacterial survival. [6] The mechanism of action of Mur ligases (MurC, D, E, F) has been studied through kinetic experiments, site-directed mutagenesis, and X-ray crystallography. [7–21] These studies have shown that the Mur ligases share the same reaction mechanism (Fig 1A), which consists first of the activation of the carboxyl group of the nucleotide precursor by ATP, generating an acyl phosphate intermediate and ADP; the acyl phosphate then undergoes a nucleophilic attack by the amino group of the condensing amino acid (or dipeptide), leading to the formation of a high-energy tetrahedral intermediate, which eventually breaks down into amide or peptide and phosphate. In addition, it has been shown that MurC and MurF follow a strictly ordered kinetic mechanism in which ATP binds first, followed by the nucleotide substrate and then the condensing amino acid or dipeptide. [7–13]

From a structural point of view, Mur ligases are three-domain enzymes that share a very well conserved ATP-binding site that harbors the Gly-X-X-Gly-Lys-Thr/Ser [12, 14] motif and display notable structural similarities in terms of domain arrangement. [20–21] The N-terminal domain is involved in the binding of the UDP-precursor, the central domain in the binding of ATP, and the C-terminal domain in the binding of the amino acid or dipeptide. [9, 19–21] Crystal structures of Mur ligases reveal that MurC, MurD and MurF display at least two distinct conformations, namely ‘open’ and ‘closed’, with the position of C-terminal domain dictating the nature of the conformation. [20–21] This has led to the suggestion that formation of an active catalytic site requires closing of the structure upon substrate binding. [7]

MurD catalyzes the addition of D-glutamic acid to UDP-Mur*N*Ac-L-Ala, generating the dipeptide moiety L-Ala-D-Glu in almost all bacterial species. [22] As mentioned above, structural information on *E*. *coli* MurD reveals two open conformations: an unbound one and an UDP-*N*-acetylmuramoyl-L-alanine (UMA)-complexed one. [10] In addition, several structures of substrate- or product-bound closed conformations have been published. [8–9, 16] *E*. *coli* MurD has been the object of targeted molecular dynamics simulation studies that have shed light on the conformational changes that occur upon ligand binding as presented in Fig 1B. These studies have revealed that upon binding of ATP and UMA, there is a ‘closing’ rotation of the C-terminal domain, which does not occur before ATP is bound. [23] *E*. *coli* MurD has been the object of extensive studies aiming at the development of new inhibitors, [24–43] but only a limited number of ligands could be crystallized within the MurD active site, [16, 30, 34, 37, 40, 42–43] or were shown to interact with MurD through NMR experiments. [38, 44–45] In all solved X-ray structures of MurD with inhibitors, the ligase is in closed conformation. Notably, the exploitation of this tractable, potential antibiotic development target requires a precise knowledge of the structural modifications engendered by ligand recognition. In order to structurally characterize the different conformational changes of MurD in atomic detail, we solved two novel crystal structures of *E*. *coli* MurD either in the presence or in the absence of ligands. This work led us to identify novel conformational properties of MurD involving an intermediate conformation in the presence of ADP and UMA, as well as an intermediate conformation in the absence of ligands. This work thus reveals that substrate binding is not the strict causative agent of domain closure, and that Mur enzymes display a variable amount of flexibility, both in the presence and absence of substrates, which could be essential for their activity in the cell. In addition, our structural analyses indicate that the kinetic mechanism of MurD, which had previously been suggested as being ordered by similarity with MurC and MurF [7, 13] may in fact be distinct, since protein function could be potentially affected by domain flexibility.

## Materials and Methods

### Crystallization and data collection

MurD was expressed and purified using the 6×His-tag system described. DH5α cells harbouring the pABD16/MurD vector were grown in 2-YT medium containing ampicillin (100 μg/mL) at 37°C in a rotary shaker to reach A600nm of 3.5. Expression was induced by adding isopropyl β-D-thiogalactopyranoside at a final concentration of 1 mM and growth was continued overnight at 20°C. The cells were lysed by sonication and the 6×His-tagged protein was purified by affinity binding to a Ni^2+^-nitrilotriacetate-agarose column and elution with a discontinuous gradient of imidazole. The enzyme was recovered in the 100 mM fraction. It was dialysed against 20 mM Hepes (pH 7.4), 200 mM NaCl, 5 mM DTT, 0.05% (w/v) NaN_3_. The amount of protein obtained was determined by the Bradford method, quantitative amino acid analysis, and by measuring the absorbance at 280 nm. The purity of the protein was checked by SDS-PAGE and MALDI-TOF mass spectrometry. [16] Both conformations crystallized in an orthorhombic space group with different cell parameters and contained one molecule per asymmetric unit (Table 1).

**Table 1:** X-ray diffraction data and structure refinement.

DATA COLLECTION		
Data set	*Intermadiate Free MurD*	*Intermadiate Bound MurD*
X-ray source	BM14U	ID29
Detector	MARCCD 225	ADSC Q315R
Wavelength (Å)	0.976	0.976
Scan-range (°)	120	180
Oscillation (°)	1	1
Space group	P2_1_2_1_2_1_	P2_1_2_1_2_1_
Cell parameters *a*, *b*, *c* (Å), α = β = γ = 90°	58.12, 70.43, 100.58	66.44, 89.84, 108.54
Mosaicity (°)	0.270	0.134
Resolution (Å)	1.84 (1.95–1.84)	1.90 (2.01–1.90)
No. observed/unique reflections	145065/34760	280913/51622
Completeness (%)	95.1 (96.3)	98.2 (94.8)
R_*sym*_ (last shell)	6.4 (49.8)	6.4 (61.6)
*I/*σ*(I)* (last shell)	22.74 (3.01)	23.75 (3.22)
Wilson plot B factor (Å^2^)	26.88	37.01
**MOLECULAR REPLACEMENT**		
Mol/ASU	1	1
Phaser LLG	3761	4284
**REFINEMENT**		
R_*work*_/R_*free*_ (%)	18.94/23.07	19.81/23.23
RMS deviation, bond lengths (Å)	0.010	0.011
RMS deviation, bond angles (°)	1.538	1.492
Mean B factor (Å^2^)	14.96	21.55
N-terminal domain mean B factor (Å^2^)	13.52	19.74
Central domain mean B factor (Å^2^)	12.97	19.29
C-terminal domain mean B factor (Å^2^)	13.20	19.95
SO_4_ mean B factor (Å^2^) / No. of	43.07 / 6	—
UMA mean B factor (Å^2^)	—	30.77
ADP mean B factor (Å^2^)	—	22.72
No. of protein/water atoms	3259/297	3285/269
Residues in most favored/allowed region of Ramachandran plot (%)	100	100

Crystallization of the intermediate conformation of ligand free MurD (*Intermediate Free MurD*):

We scanned various crystallization conditions in order to obtain different forms of MurD crystals, which could then be soaked with different inhibitor solutions. Different combinations of buffers, precipitants, co-precipitants, pH values and crystallization temperatures were tried in the presence or absence of MurD substrates. Several different forms of crystals grew over the time and after solution of all of the structures, new Intermediate Free MurD and Intermediate Bound MurD structures were identified. Concurrently, as a positive control, the crystals of open [10] and closed [8, 9] forms of MurD were also produced using the crystallization conditions described previously.

*Intermediate Free MurD* was crystallized by mixing 2 μL of protein sample (3 mg/mL, in 20 mM HEPES, pH 5.6, 1 mM DTT, and 1 mM NaN_3_) and 2 μL of reservoir solution (1.8 M (NH_4_)_2_SO_4_, 7% (v/v) (±)-2-methyl-2,4-pentanediol and 0.1 M MES, pH 5.6) at 15°C by vapor diffusion using the hanging-drop method. Crystals grew in 48 hours. X-ray diffraction data were collected at the European Synchrotron Radiation Facility (ESRF, Grenoble, France) (Table 1).

Crystallization of intermediate conformations of MurD with ligands (*Intermediate Bound MurD*):

Crystals of *Intermediate Bound MurD* were obtained at 15°C by vapor diffusion using the hanging-drop method. Crystals were grown by mixing 2 μL of protein (4 mg/mL, 1 mM UMA, 5 mM AMP-PNP, 1 mM NaN_3_, 1 mM DTT, and 20 mM HEPES, pH 7.4) with 2 μL of reservoir solution (1.8 M Na-malonate, pH 7.0). Crystals appeared in 6 months and data was collected at the ESRF, as above (Table 1).

### Structure determination and refinement

X-ray diffraction data sets were indexed and scaled with XDS. [46] The structure was solved by employing the structure of MurD of *E*. *coli* (PDB entry: 3uag [9]) split into two search models (model 1: N-terminal and central domains (residues 1–299); model 2: C-terminal domain (residues 300–437)) in a molecular replacement approach using PHASER. [47] The solution model was rebuilt *de-novo* in order to reduce bias from the model using ARP/wARP. [48] COOT [49–51] was used for manual corrections of the model. Cycles of refinement employing three domains by TLS definition [52] (residues 1–92, 94–295 and 304–439) were performed by REFMAC 5.5 [53] as implemented in the CCP4-6.3.0 suite of programs. [53] In addition, water molecules were added to the residual electron density map using ARP/ wARP. [48] After several cycles of manual model building and refinement, R_work_ and R_free_ converged. The stereochemical quality of the refined model was verified with MOLPROBITY [54] and PROCHECK. [55] The secondary structure assignment was performed by DSSP [46] and STRIDE. [56] RMSD values for the best fit for the whole protein, separate domains, rotation angle and translation of C-terminal domain and bending region analysis were calculated using DynDom server. [57] For identification of protein-ligand interactions and interactions between amino acid residues, LIGPLOT [58] and LigPlot+ [59] were used. X-ray diffraction data, structure solution and refinement statistics are found in Table 1. Figures containing protein structures were generated with PyMol. [60]

## Results

Structures of *E*. *coli* MurD solved by Bertrand and coworkers either in unbound form or in the presence of substrates or products suggest that ligand binding engenders a conformational movement of the C-terminal domain towards the center of the structure, thus leading to the closure of the enzyme. [9–10] Since the detailed knowledge of all of the forms of MurD is essential for optimal exploitation of this enzyme for potential antibiotic development, we initiated a study on the different conformations of *E*. *coli* MurD, initially by attempting to trap MurD in states that were distinct from those which had been previously described. We achieved this by solving two intermediate conformations of MurD which we named *Intermediate Free MurD* (*i*.*e*., intermediate conformation of ligand-free MurD) and *Intermediate Bound MurD* (*i*.*e*., intermediate conformation of MurD with ligands).

Results regarding these structures are presented in three separate sections in which *i) Intermediate Free MurD* and *ii) Intermediate Bound MurD* are both compared to the previously solved structures of MurD, and *iii)* similarities and differences between the two new conformations of MurD are highlighted.

### Intermediate Free MurD

In the solving of *Intermediate Free MurD*, 421 residues were refined. There is one minor region missing between residues Thr180─Tyr187 since it could not be traced in the electron density map. Notably, Lys198 is not carbamoylated as it is the case in open MurD structures.^7^

**Fig 2:**
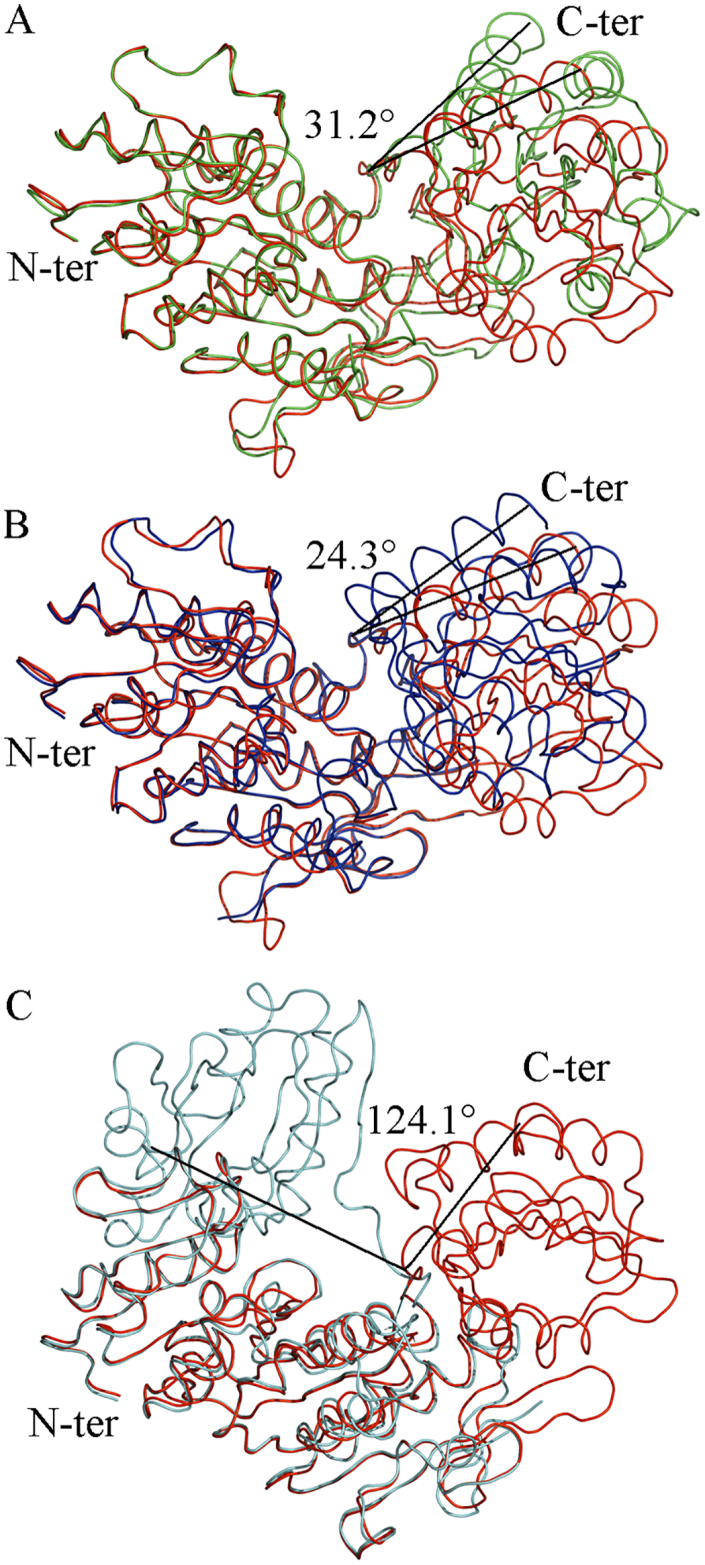
Superposition of *Intermediate Free MurD* (red) to known MurD structures with identified angles of rotation of the C-terminal domain. (A) open MurD (green) (PDB entry: 1e0d [7]). (B) closed MurD (blue) (PDB entry: 3uag [6]). (C) open MurD (cyan) (PDB entry: 1eeh [7]).

In order to characterize our *Intermediate Free MurD* structure, we superposed it onto that of other previously solved *E*. *coli* MurD structures (Fig 2). Superposition to open unbound MurD structure [10] (Fig 2A) revealed that the new structure was clearly in a distinct conformation (Table 2). Further investigation consisted in superimposing it to the closed structure of UMA-bound MurD [9] (Fig 2B). It appeared that it was not in a closed conformation. After superposition of *Intermediate Free MurD* with open UMA-bound MurD [10] (Fig 2C), we confirmed that this new conformation of MurD is different from the previously described ones. These analyses revealed that, in spite of the absence of ligands, our new structure was not completely open, and rather displayed an intermediate conformation. This fact was quantified by the determination of the RMSD values of all of the superimposed structures (Table 2), both for the whole protein and for distinct domains.

**Table 2:** Analysis of the superposition of the *Intermediate Free MurD* and *Intermediate Bound MurD*.

Aligned structures	Overall^a^	N-ter-central^b^	C-ter^c^	^d^ (°)	^e^(Å)	Residue
3uag/*Free*	3.12 [417/421]	0.98 [276/293]	0.41 [141/144]	24.3	-1.8	298–301
1e0d/*Free*	3.70 [421/421]	1.21 [282/294]	0.43 [141/143]	31.2	1.5	298–301
1eeh/*Free*	15.33 [419/421]	1.06 [278/294]	1.32 [141/143]	124.1	-4.6	297–304
3uag/*Bound*	3.69 [420/423]	0.84 [279/294]	0.43 [141/144]	30.0	-0.7	298–300
1e0d/*Bound*	3.78 [419/423]	0.99 [287/294]	0.56 [141/143]	33.9	1.0	297–301
1eeh/*Bound*	15.21 [419/423]	0.64 [278/294]	1.40 [141/143]	123.4	-3.0	296–304
*Free* /*Bound*	1.30 [416/421]	1.01 [277/294]	0.65 [139/143]	7.5	0.2	118–122, 234–262, 291–292

^a^ RMSD (Å) [No. Cα] of the two superimposed structures best fit

^b^ RMSD (Å) [No. Cα] of the fixed domains of the two superimposed structures best fit. Here N-terminal and central domains are seen as one non-rotating domain

^c^ RMSD (Å) [No. Cα] of the rotating C-terminal domain of the two superimposed structures best fit

^d^ Rotation of the C-terminal domain around the inter-domain screw axis relative to central and N-terminal domain

^e^ Translation along the inter-domain screw axis. The minus sign represents translation in opposite direction along of the inter-domain screw axis

**Fig 3:**
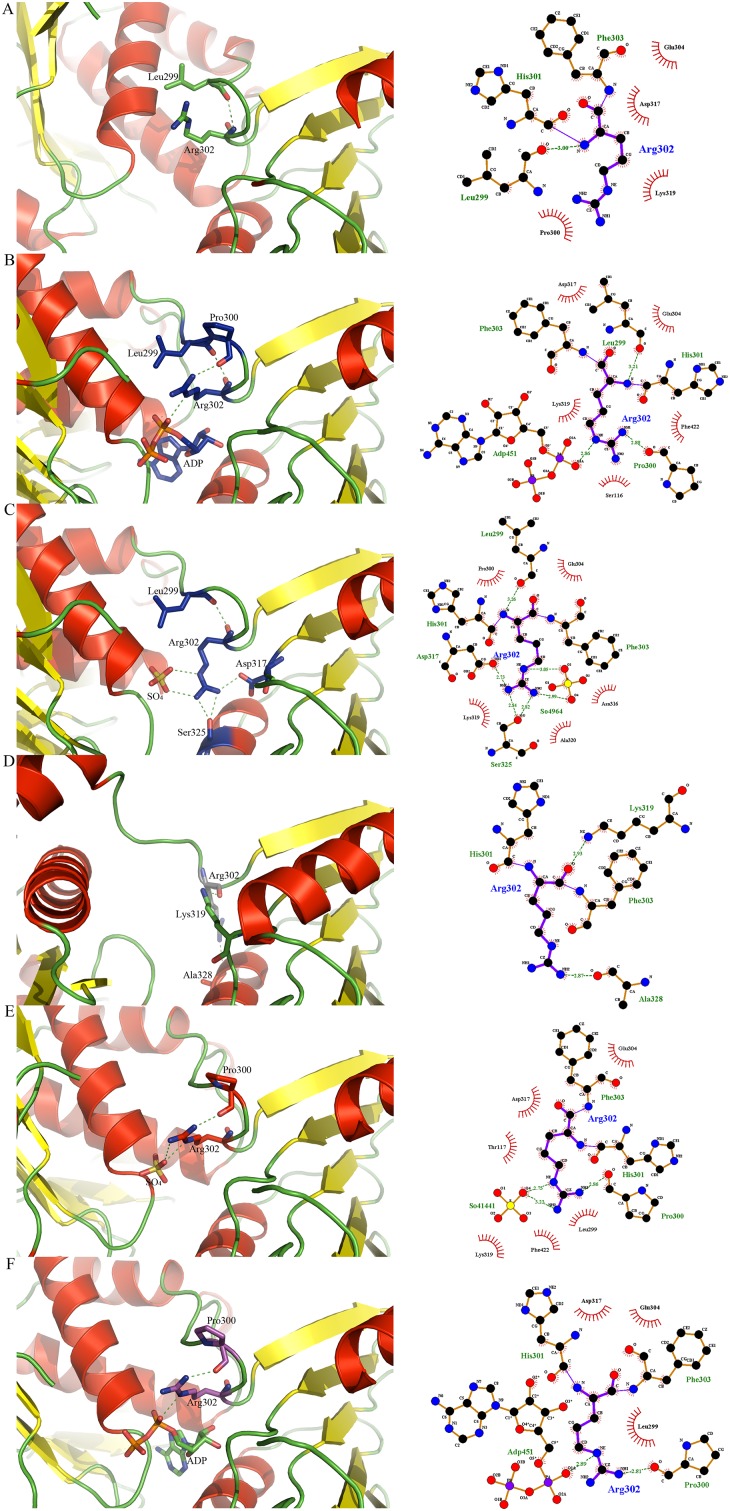
Orientation and LIGPLOT representations of Arg302 in MurD structures. (A) Open MurD (PDB entry: 1e0d [7]). (B) Closed ADP- and UMA-bound MurD (PDB entry: 3uag [6]). (C) Closed UMA-bound MurD (PDB entry 1uag [5]) (D) Open UMA-bound MurD (PDB entry 1eeh [7]). (E) *Intermediate Free MurD*. (F) *Intermediate Bound MurD*.

We analyzed the conformations of key residues that contribute to the binding of UMA [9–10] and discovered that they are the same as in closed UMA-bound MurD structures [9] We also compared the orientations of residues that contribute to ADP binding and found that they share basically the same conformation as previously reported. [9, 16] Only Arg302 was found to be differently oriented so we focused on this residue because of its position in the hinge loop containing residues from Thr294 to Glu304. There is a difference in the orientation of Arg302 as well as its interactions, when compared to the previously solved closed conformations of MurD (Fig 3A–3D) and additionally orientation of Arg302 in *Intermediate Free MurD* is different from all of the orientations found in the aforementioned structures of MurD (Fig 3E), which contributes towards the intermediate conformation adopted by the protein.

### Intermediate Bound MurD

**Fig 4:**
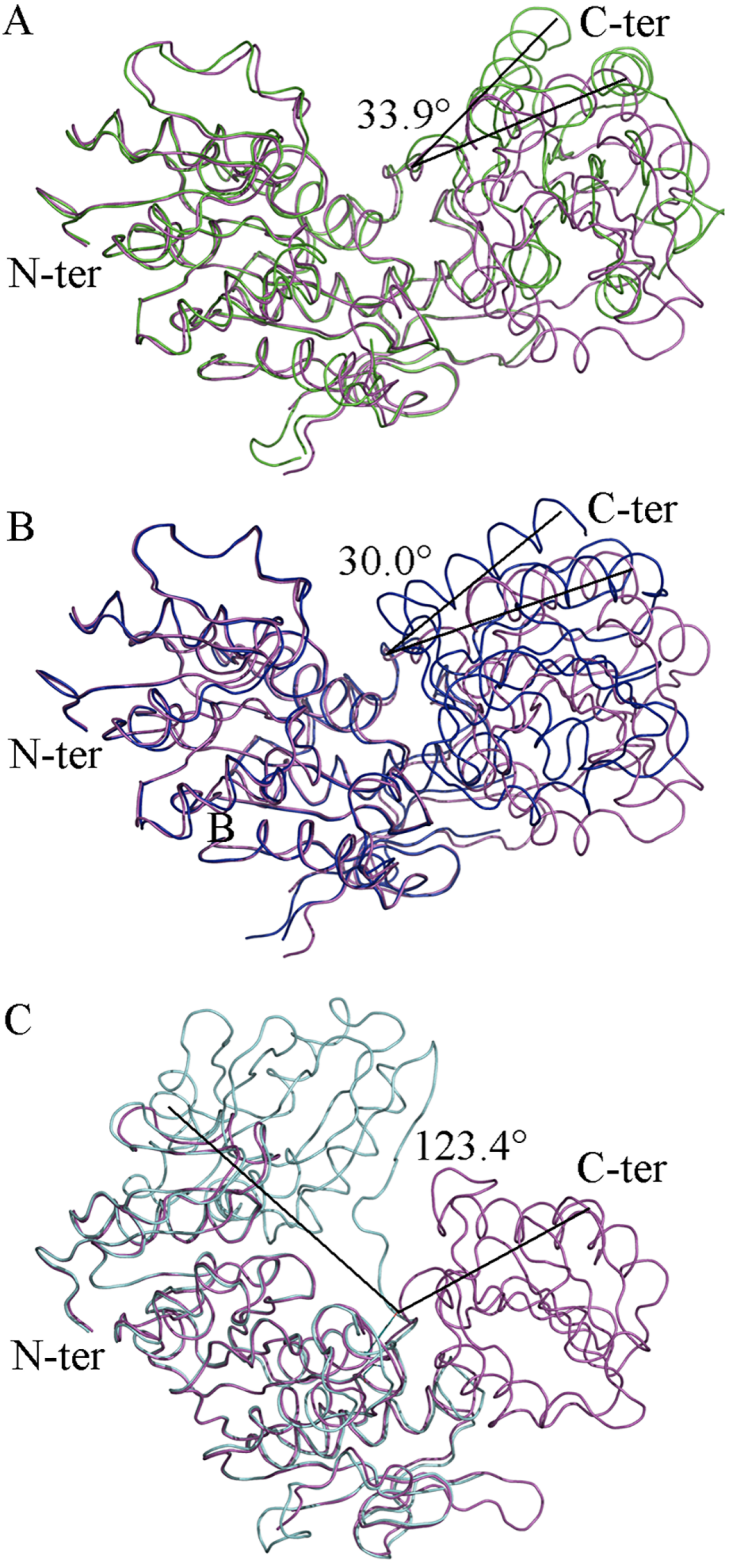
Superposition of *Intermediate Bound MurD* (magenta) to known MurD structures structures with identified angles of rotation of the C-terminal domain. (A) open MurD (green) (PDB entry: 1e0d [7]). (B) closed MurD (blue) (PDG entry: 3uag [6]). (C) open MurD (cyan) (PDB entry: 1eeh [7]).

*Intermediate Bound MurD* was co-crystallized with UMA and AMP-PNP in order to further explore the dynamics of MurD upon ligand binding. During the process of structure solution, 423 residues were refined. Structural inspection of *Intermediate Bound MurD* revealed that Lys198 is not carbamoylated, contrary to all previously published structures of MurD complexed to UMA [8–9, 16] Previously, complex formation between MurD and UMA, [8] UDP-*N*-acetylmuramoyl-L-alanyl-D-glutamic acid (UMAG), [9] UMA-ADP, [13] UMA-ADP-Mg^2+^ [9] and UMA-ADP-Mn^2+^ [9] locked MurD in a closed structure, which gave weight to the proposal that ligand binding was important for the movement of the C-terminal domain leading to closure. [8–9] We investigated our *Intermediate Bound MurD* structure by superimposing it to the previously solved structures (Fig 4). Superposition to the open unbound MurD structure [10] (Fig 4A) revealed that the new structure was clearly in a distinct conformation (Table 2). Further investigation consisted in superimposing it to the closed structure of UMA-bound MurD [9] (Fig 4B). It appeared that it was not in a closed conformation. After superposition of *Intermediate Bound MurD* with open UMA-bound MurD [10] (Fig 4C), we confirmed that this new conformation of MurD is different from the previously described ones, which was quantified by the determination of the RMSD values (Table 2) of all of the superimposed structures, both for the whole protein and for distinct domains.

In the *Intermediate Bound MurD* structure, the H-bond pattern of UMA binding is almost the same as in the closed structures of MurD [8–9, 16] and as in open UMA-bound MurD. [10] ADP-binding residues also occupy the same orientations as in previously solved structures. [9, 16] When focusing on the interactions and position of Arg302 in *Intermediate Bound MurD* (Fig 3F), we observed a novel H-bond pattern for Arg302.

### Intermediate Free MurD vs. Intermediate Bound MurD

**Fig 5:**
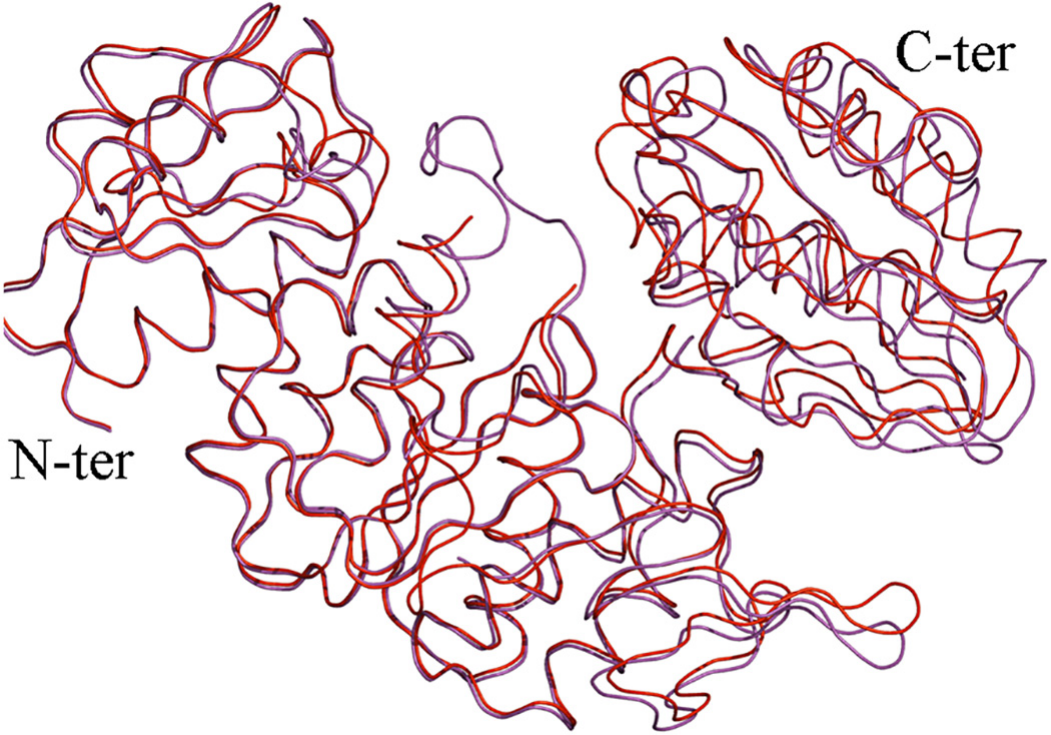
Superposition of *Intermediate Free MurD* (red) and *Intermediate Bound MurD* (magenta).

Our newly solved intermediate structures of MurD were superimposed (Fig 5). Objectively we observed that our new structures share a similar conformational space with or without ligands, which was previously not considered. The Low RMSD value of best fit for the whole protein and low RMSD values for the best fits of individual non-rotating N-terminal and central domains and C-terminal domains (Table 2), quantitatively support our claim of conformational similarities between the *Intermediate Free MurD* and *Intermediate bound MurD*.

## Discussion and Conclusions

*E*. *coli* MurD has been studied in detail at the atomic level with several X-ray structures. [9–10, 16] Lately, a paradigm has been proposed that 3D structures of proteins should be represented as an ensemble of structures. [61] Therefore, obtaining structures of proteins in distinct conformations is very important for capturing a representative subset of true native conformations. [62] Flexible regions described as “liquid-like” are the main reason for distinct conformations of proteins. Rigid regions in proteins described as “solid-like” do not contribute to distinct conformations, which again suggests that a static structure of a protein is just one of the subset of native conformations. [63] A key event in MurD catalytic activity is the C-terminal domain rigid body rotation, thought to be triggered by the binding of ATP and UMA which together create the acyl phosphate intermediate. The tetrahedral intermediate is then generated upon D-Glu binding and is eventually disrupted by the release of P_i_ and UMAG. [6, 22] From the previously discovered MurD X-ray structures, two distinct conformations, open and closed, were inferred. [10] The kinetic mechanism proposed for MurD did not consider an ATP-bound non-closed conformation. [6, 64] In the present paper we have described two new structures of *E*. *coli* MurD and compared them to all previously reported structures of unbound MurD and MurD complexed with ligands. Our analysis of different MurD conformations indicates that entire domain rotations are privileged as opposed to minor structural modifications. Thus, we were able to ‘trap’ an intermediate conformation of MurD that is not open despite the absence of ligands, nor closed in the presence of ligands. This suggests that it is not substrate recognition which causes MurD closure, as previously suggested, but that domain movement is an inherent feature of the enzyme that is independent of ligand binding.

Our discovery of new intermediate conformations of *Intermediate Free MurD* and *Intermediate Bound MurD* raises the question of what triggers C-terminal domain of MurD to adopt different conformations. Upon modification of crystallization conditions (Table 3), we managed to capture MurD in two new conformations from the ensemble of conformations that potentially exist within the bacterial cytoplasm. If we compare the conditions in which crystals were grown and the physiological cytoplasmic conditions of *E*. *coli*, we conclude that since Mg^2+^, ATP, UMA and sulfate are continuously present in bacterial intracellular space, [65–67] it would not be reasonable to expect that any conformation of MurD would be advantageous with respect to the others; most probably, there is a dynamic equilibrium of different conformational states. This suggestion is supported by computational simulations that indicate that the energy difference between open and closed conformations of MurD *in vacuum* is around *10* kcal/mol. [23]

**Table 3:** Review of *E*. *coli* MurD crystallization conditions and its conformations.

PDB entry	pH	Precipitant	Ligands	Conformation	Space group	Cell parameters
1uag	7.0	(NH_4_)_2_SO_4_	UMA	Closed	P4_1_	*a* = *b* = 65.43, *c* = 134.44 Å, α = β = γ = 90°
2uag	7.0	PEG3350	UMA, ADP, Mg^2+^	Closed	P4_1_	*a* = *b* = 65.56, *c* = 136.01 Å, α = β = γ = 90°
3uag	7.2	(NH_4_)_2_SO_4_	UMA^1^	Closed	P4_1_	*a* = *b* = 65.24, *c* = 134.41 Å, α = β = γ = 90°
4uag	7.0	(NH_4_)_2_SO_4_	UMAG	Closed	P4_1_	*a* = *b* = 65.80, *c* = 134.54 Å, α = β = γ = 90°
1e0d	6.0	(NH_4_)_2_SO_4_	-	Open	P4_3_2_1_2	*a* = *b* = 69.16, *c* = 196.71 Å, α = β = γ = 90°
1eeh	5.2	PEG3350	UMA	Open	P2_1_2_1_2_1_	*a* = 58.84, *b* = 63.28, *c* = 114.91 Å, α = β = γ = 90°
2jfg	7.5	(NH_4_)_2_SO_4_	UMA, ADP	Closed	P4_1_	*a* = *b* = 65.09, *c* = 134.25 Å, α = β = γ = 90°
*Free*	5.6	(NH_4_)_2_SO_4_	-	Intermediate	P2_1_2_1_2_1_	*a* = 58.24, *b* = 70.55, *c* = 100.75 Å, α = β = γ = 90°
*Bound*	7.0	Na-malonate	UMA, ADP	Intermediate	P2_1_2_1_2_1_	*a* = 66.44, *b* = 89.84, *c* = 108.54 Å, α = β = γ = 90°

^1^ The structure in PDB also includes ADP and Mn^2+^, which were soaked into the protein after crystals were grown in conditions in the presence of (NH_4_)_2_SO_4_ as precipitant and UMA.

However, the importance of carbamoylated Lys198 (KCX198) must not be neglected. KCX198 was only found in closed “active” conformations of MurD. [9–10] Its importance was studied by Dementin *et al*. [68], who established that KCX198 plays a role in the enzymatic reaction as it stabilizes Mg^2+^, an essential cation involved in the MurD activity, via two water molecules. Indeed, enzymatic activities of Lys198 MurD mutants were considerably less active than the native one, and the activity could be restored upon incubation with short-chain carboxylic acids (chemical rescue). [68] Therefore, for the activation of MurD, not only binding of the substrates, but also carbamoylation of Lys198 is essential. Most probably, *Intermediate Bound MurD* represents the conformation which is adopted after substrates are bound, but it is not yet in the closed “active” conformation, since Lys198 is not carbamoylated. So far, there was no evidence that carbamoylation of Lys198 is a transient event. Nevertheless, the crystal structures of open MurD (PDB ID: 1e0d and 1eeh, where Lys198 is not carbamoylated), and closed MurD (PDB ID: 1uag, 2uag, 3uag, 4uag, 2jfg, where Lys198 is carbamoylated), suggest that carbamoylation of Lys198 occurs during catalysis. Of course, the possibility of chemical degradation of KCX198 due to the long period in which the crystals grew cannot be totally ruled out.

*Intermediate Bound MurD* displays some features of the closed conformation of MurD. The hinge loop is similar to the one in open conformation of MurD, but with an additional H-bond to Pro300 and ADP, thus dictating the C-terminal domain to take a position closer to central domain than open conformation of MurD. [10] However, the C-terminal domain does not rotate to the closed conformation, because Arg302 is not H-bonded to Leu299 as it is the case in closed MurD. A common feature of the closed conformations with ligands is also the interaction of Arg302 to sulfate (Fig 3C and 3E) or α-phosphate of ADP (Fig 3B). The higher number of H-bonds between Arg302 and the residues in non-rotating central domain via ADP or sulfate result in conformations that are either closed or intermediate, while the lower number of H-bonds of Arg302 to the non-rotating domains restricts MurD into the open or intermediate conformation. This claim is supported by computational simulations that suggest that the energy oscillations during the transition from open to closed state *in vacuum* were on average between *10* and *15* kcal/mol. [23] The estimated energy is *5*–*6* kcal/mol for the bond *in vacuum* and *0*.*5*–*1*.*5* kcal/mol for the bond in aqueous solution, [69] which is approximately the estimated energy needed to induce C-terminal domain rotation, but probably not enough to fully lock it in the closed conformation. These estimations for the H-bond energy are calculated for water-free protein, but the energy of the H-bond in solvated proteins is 2- to 3-fold lower, [69] which gives even more weight to the assumption that H-bonding provides sufficient energy for conformation changes if the bond is formed or broken. [70] With total count of H-bonds and their energy in mind, Arg302 can be described as a molecular handle, which enables the C-terminal domain of MurD to rotate.

Another aspect of the biology of Mur ligases is their assembly inside the bacterial cytoplasm. It has been speculated that Mur ligases are assembled *in vivo* in the form of a multi-enzyme complex; [71] however, no evidence of the existence of such a complex between Mur ligases has been provided to date. Instead, interactions with other proteins involved in peptidoglycan synthesis (MurI, DdlA, MurG) or cell division (MreB) have been reported in *Caulobacter crescentus* [72], *Mycobacterium tuberculosis* [73] and *Thermotoga maritima* [15]. In the latter species, it was shown with surface plasmon resonance experiments that MurD, MurE and MurF interact with MurG and MreB. These experiments were performed in ligand-free environment, thus it was speculated that these two enzymes could act as scaffolds to maintain Mur ligases assembled to each other even in their open, inactive conformation. [15] Whether contact with these or other proteins affects the modifications of conformation we have observed is currently not known. Such a problem will be understood only by solving the structure of protein complexes in which Mur ligases are involved.

The kinetic mechanism of the Mur ligases was firmly established in the cases of MurC [13] and MurF [7] It was shown to be strictly ordered, with ATP binding first, followed by the UDP-precursor and, finally, the amino acid (or dipeptide). By similarity, the same mechanism was inferred for MurD, an assumption that was supported by the discovery of open and closed conformations. [7] However, our present structural data, which reveal the existence of an ATP-bound, open conformation, indicate that the actual kinetic mechanism of MurD may be distinct. This is in keeping with the observation that the molecular isotope exchange reaction is not strictly ADP-dependent [74], contrary to MurC, [75] which suggests some randomness in the kinetic mechanism.

**Fig 6:**
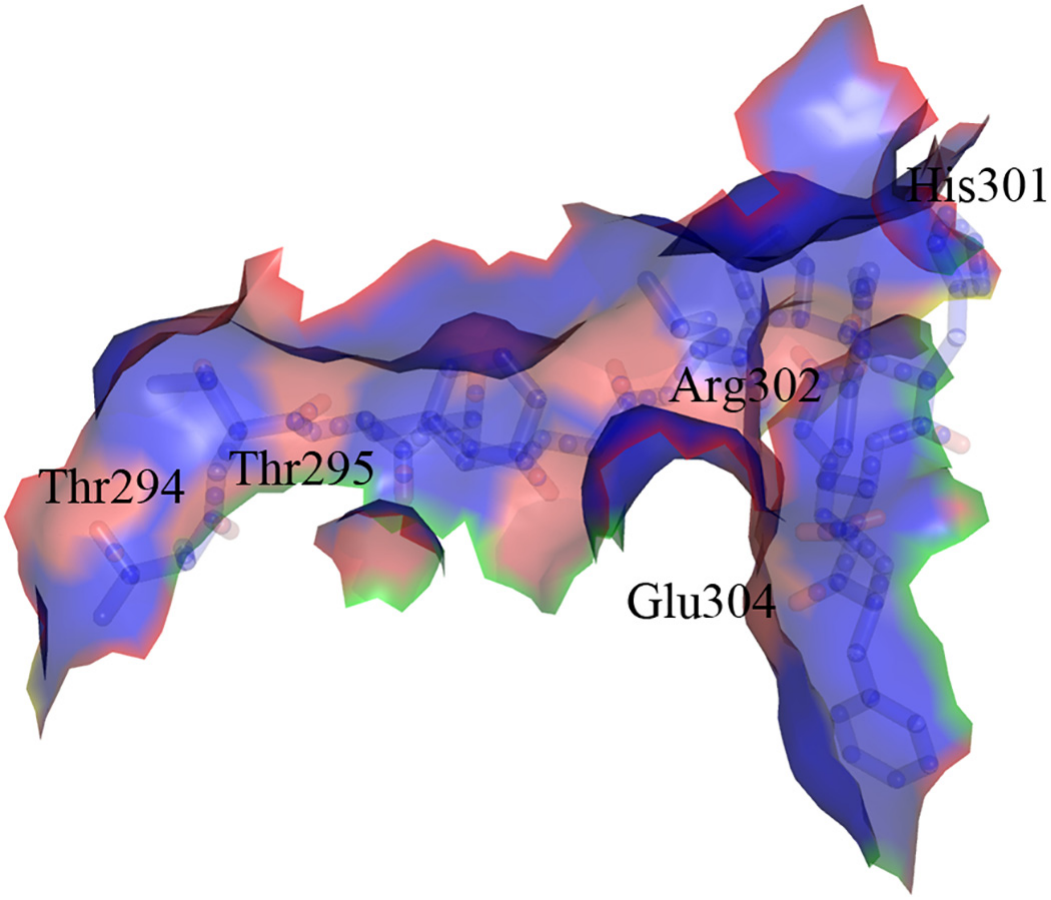
Hinge loop containing residues Thr294−Glu304. Potential target for new allosteric MurD inhibitors. Red regions represent the potential targets for H-bond interactions, blue regions represent hydrophobic surface of the hinge loop.

The crystal structures presented in this paper provide us with new tools in the urgent mission to discover novel antibacterial agents. So far, inhibitors of MurD that were designed to bind in the substrate-binding sites have not expressed significant antibacterial activity. [6, 22] Taking into account the detailed knowledge about the ability of MurD to take distinct conformations, new inhibitors can be designed not only as mimics of the natural MurD substrates, but also as smaller allosteric inhibitors. A region which could potentially be targeted by allosteric inhibitors is the hinge loop (Fig 6), which is involved in the C-terminal domain rotation. Thr294, Thr295, His301, Arg302 and Glu304 could be specifically targeted for potential polar interactions with inhibitors due to the presence of several lone electron pairs in their side chains. Structure-based designed allosteric inhibitors with affinity towards this loop could either prevent the binding of substrates or freeze the enzyme in the conformation where domain rotation and consequently catalytic activity are prevented.

### Protein Data Bank accession numbers

Intermediate conformation of ligand free MurD (*Intermediate Free MurD*): 5a5e (S1 Checklist).

Intermediate conformation of MurD with ligands (*Intermediate Bound MurD*): 5a5f (S2 Checklist).

## Supporting Information

S1 Checklist Protein Data Bank Validation Reports 5a5e. (PDF)

S2 Checklist Protein Data Bank Validation Reports 5a5f. (PDF)
